# Unraveling Perinatal Risks in Small-for-Gestational-Age vs. Adequate-for-Gestational-Age Fetuses: Is a Pathological Cerebroplacental Ratio a Red Flag for All?

**DOI:** 10.7759/cureus.92450

**Published:** 2025-09-16

**Authors:** Arya J, Chandrashekhar Shrivastava, Sarita Agrawal, Sarita Rajbhar, Atiya Raza

**Affiliations:** 1 Obstetrics and Gynecology, All India Institute of Medical Sciences, Raipur, Raipur, IND

**Keywords:** adequate for gestational age (aga), apgar score, cerebroplacental ratio (cpr), fetal growth restriction (fgr), neonatal resuscitation, perinatal outcomes, small for gestational age (sga), umbilical cord ph

## Abstract

Introduction

The association between pathological cerebroplacental ratio (CPR) and perinatal outcomes has garnered significant attention in obstetric research, particularly with regard to fetal growth patterns. This study aims to explore the implications of pathological CPR in small-for-gestational-age (SGA) fetuses compared with those classified as adequate-for-gestational-age (AGA). In clinical practice, CPR assessment has gained prominence due to its potential to improve decision-making regarding the timing and mode of delivery in pregnancies complicated by growth restriction. Previous research has shown that an abnormal CPR may indicate compromised fetal well-being, necessitating closer surveillance and intervention. However, several studies have also demonstrated that CPR has limited predictive performance when used as a routine screening tool for adverse perinatal outcomes at 36 weeks' gestation. This prospective observational cohort study seeks to provide further insight into the relationship between pathological CPR and perinatal outcomes in AGA and SGA fetuses, thereby contributing to improved maternal-fetal care.

Objective

The objective of this study is to assess and compare perinatal outcomes in SGA and AGA fetuses with pathological CPR, with a focus on delivery characteristics and early neonatal complications.

Methods

A prospective cohort study was conducted over 18 months at the All India Institute of Medical Sciences, Raipur, enrolling 80 pregnant women (>36 weeks’ gestation) with pathological CPR. Participants were categorized as SGA (<10th percentile, n = 40) or AGA (>10th percentile, n = 40) based on estimated fetal weight. Weekly follow-ups included clinical assessments and Doppler studies, with management guided by the Barcelona protocol. Outcomes were recorded at delivery and during the first week of neonatal life.

Results

SGA fetuses (n = 40, 50%) were delivered at an earlier gestational age (37.68 ± 1.15 vs. 38.3 ± 0.99 weeks; p < 0.05) and more frequently within seven days of diagnosis (31/40 (77.5%) vs. 20/40 (50%)). Caesarean section due to fetal distress was significantly more common in the SGA group (18/21 (85.71%) vs. 9/19 (47.37%); p = 0.017), while failed induction occurred more often in AGA fetuses (6/19 (31.58%) vs. 1/21 (4.76%); p = 0.04). SGA neonates had lower APGAR scores at five and 10 minutes and lower mean cord pH (p = 0.003). NICU admissions (13/39 (33.33%) vs. 10/40 (25%)), respiratory distress, and early neonatal deaths were also more common in the SGA group, although not all differences reached statistical significance.

Conclusions

Pathological CPR in SGA fetuses is associated with earlier delivery and poorer neonatal outcomes compared with AGA fetuses, despite similar management. These findings underscore the importance of incorporating CPR assessment into fetal surveillance while avoiding unnecessary interventions in AGA fetuses with pathological CPR.

## Introduction

The cerebroplacental ratio (CPR), defined as the ratio of the middle cerebral artery (MCA) to umbilical artery (UA) pulsatility index, is an emerging predictor of adverse pregnancy outcomes in both small-for-gestational-age (SGA) and adequate-for-gestational-age (AGA) fetuses [1]. SGA is defined as a fetal weight below the 10th percentile, whereas fetal growth restriction (FGR) refers to a failure to achieve genetic growth potential [1]. In clinical practice, CPR assessment has gained prominence due to its potential to inform decision-making regarding the timing and mode of delivery in pregnancies complicated by growth restriction [2,3].

Previous research has indicated that an abnormal CPR may serve as a marker of compromised fetal well-being, necessitating closer surveillance and intervention. However, several studies have also reported that CPR demonstrates limited predictive performance when used as a routine screening tool for adverse perinatal outcomes at 36 weeks’ gestation [4]. Late-onset FGR often presents with normal UA Doppler findings despite placental damage of less than 30%, making detection before 36 weeks difficult [5]. While SGA is typically associated with early-onset preterm stillbirths, AGA fetuses account for most term stillbirths [6,7]. Growth-restricted fetuses with biometric measurements above the 10th percentile may fail to reach their genetic growth potential and therefore remain undiagnosed, despite being at increased risk of adverse outcomes.

CPR is more sensitive to hypoxia than UA or MCA alone, as it declines in response to brain-sparing effects during fetal hypoxia. A CPR below the fifth percentile is considered pathological and is associated with an increased risk of labor distress and poor neonatal outcomes [1]. The Barcelona criteria (DELPHI consensus) classify FGR into four stages based on estimated fetal weight and Doppler parameters, guiding surveillance and delivery timing, with stage 1 FGR generally recommended for delivery at 37 weeks [5].

This study compares adverse perinatal outcomes in SGA versus AGA fetuses with pathological CPR after 36 weeks. Evidence suggests that low CPR, regardless of fetal size, is associated with an increased risk of operative delivery, low neonatal pH, and NICU admission, particularly when assessed within two weeks of delivery [7,8]. The objective is to evaluate whether AGA fetuses with low CPR warrant the same level of clinical attention as SGA fetuses, thereby underscoring the potential value of incorporating routine third-trimester CPR assessment into standard antenatal screening and induction-of-labor protocols.

## Materials and methods

Study design and setting

After obtaining ethical clearance, this prospective study was conducted from May 1, 2023, to October 31, 2024, at the All India Institute of Medical Sciences (AIIMS), Raipur. Pregnant women beyond 36 weeks of gestation with pathological CPR, attending routine outpatient or inpatient antenatal clinics, and meeting the inclusion and exclusion criteria were recruited. Participants were assigned to two groups based on estimated fetal weight at the time of recruitment: AGA (estimated fetal weight >10th percentile but <90th percentile) and SGA (estimated fetal weight <10th percentile according to Hadlock charts).

Ethical considerations and consent

The study protocol was explained to all participants through a patient information sheet, and written informed consent was obtained. Participant history, demographics, clinical findings, and gestational age at the first diagnosis of pathological CPR were recorded. Pathological CPR was defined as a CPR below the fifth percentile for gestational age, corresponding to a value <1.08, in accordance with established protocols, calculated using the Barcelona protocol. Prior investigations were documented in a structured case record form.

Follow-up and monitoring

Participants were followed weekly from 36 weeks of gestation, in accordance with standard protocol. Each visit included assessment of maternal vitals, fetal parameters, Doppler studies, and obstetric ultrasonography for amniotic fluid index and Doppler measurements at recruitment and as indicated thereafter. To minimize inter-observer variability, all ultrasounds were performed by the same FMF UK-certified co-investigator using the same ultrasound machine (MINDRAY Z6) following ISUOG guidelines [9] at the Obstetrics and Gynecology OPD, AIIMS, Raipur.

During follow-up, patients with complications such as antepartum hemorrhage, severe preeclampsia, eclampsia, insulin-dependent gestational diabetes mellitus, or other conditions requiring early delivery were excluded. Fetal parameters were assessed for worsening FGR at each visit and managed according to the Barcelona protocol. Participants were followed until delivery, and neonatal outcomes were tracked for seven days postpartum.

Inclusion and exclusion criteria

Pregnant women aged >18 years with a singleton pregnancy beyond 36 weeks of gestation, diagnosed with pathological CPR, and providing informed consent were included. Exclusion criteria comprised the presence of fetal congenital anomalies or chromosomal abnormalities, early-onset FGR diagnosed before 32 weeks, medical disorders necessitating immediate termination of pregnancy (e.g., placenta previa, abruption, and eclampsia), and evidence of ductus venosus wave reversal or absent end-diastolic flow in the UA. Multiparous women were also excluded to avoid potential confounding effects on obstetrical outcomes, including mode of delivery.

Sample size calculation

With reference to the study by DeVore [1], considering the prevalence of pathological CPR in SGA fetuses as 39.3% and in AGA fetuses as 11%, the sample size was calculated using the formula:

\[N = \frac{(Z_{1-\alpha/2} + Z_{1-\beta})^2 \, (P_1 q_1 + P_2 q_2)}{d^2},\]

where Z₁₋α/2 = 1.96, the value of the standard normal distribution for a two-sided test at the 0.05 significance level; Z₁₋β = 0.84, corresponding to 80% power; P₁ = 0.393, the prevalence of pathological CPR in SGA fetuses; P₂ = 0.11, the prevalence of pathological CPR in AGA fetuses; q₁ = 1 − P₁, q₂ = 1 − P₂; and d = P₁ − P₂.

Substituting the values, the required sample size per group was calculated as

\[N = \frac{(1.96 + 0.84)^2 \times (0.39 \times 0.61 + 0.11 \times 0.81)}{0.28^2} \approx 33\]

Thus, 33 participants were required in each group, giving a total of 66. After accounting for a 20% dropout rate, the final adjusted sample size was 40 per group, resulting in a total of 80 participants.

Statistical analysis

**Figure 1:**
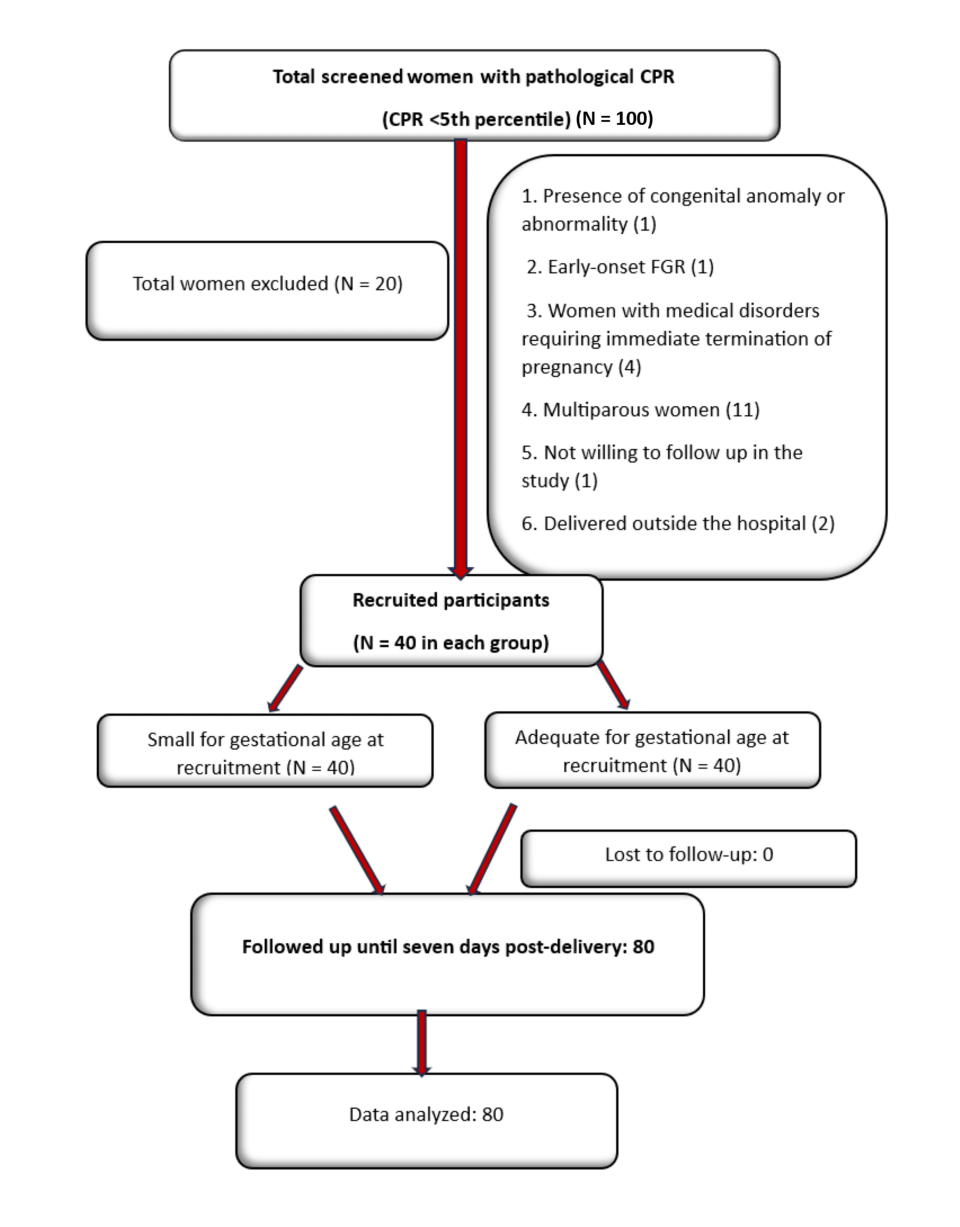
Study methodology flow chart (STROBE diagram) CPR, cerebroplacental ratio; FGR, fetal growth restriction; STROBE, STrengthening the Reporting of OBservational studies in Epidemiology

The numerical values and percentages were used to summarize categorical variables, while quantitative data were presented as medians with interquartile ranges (25th-75th percentiles) and as means ± SD. The Shapiro-Wilk test was applied to assess data normality, and non-parametric tests were used when the data were not normally distributed. The Mann-Whitney test was used for quantitative variables that were not normally distributed, and the independent t-test was applied for normally distributed quantitative variables. The chi-squared test was used to compare qualitative variables, with Fisher’s exact test applied when any expected cell value was less than 5. The RR of various adverse outcomes in SGA fetuses compared with AGA fetuses was also calculated. Data were entered into Microsoft Excel (Microsoft Corporation, Redmond, WA, USA) and analyzed using IBM SPSS Statistics for Windows, Version 25.0 (Released 2017; IBM Corp., Armonk, NY, USA), and a p-value of less than 0.05 was considered statistically significant. The study methodology is illustrated in Figure 1, which outlines the enrollment, follow-up, and analysis of study participants (STrengthening the Reporting of OBservational studies in Epidemiology (STROBE) diagram).

## Results

This study compared labor and neonatal outcomes between SGA and AGA fetuses with abnormal CPR. Comparable rates of vaginal delivery and emergency lower segment cesarean section were observed in both groups (Table 1). Analysis of intrapartum cardiotocography (CTG) findings demonstrated a statistically significant difference between groups (p = 0.003).

**Table 1:** Comparison of mode of delivery between SGA and AGA AGA, adequate for gestational age; LSCS, lower segment cesarean section; SGA, small for gestational age

Mode of birth	SGA (n = 40)	AGA (n = 40)	Total (n = 80)	p-Value (Fisher’s exact test)
Vaginal delivery	18 (45%)	17 (42.5%)	35 (43.75%)	0.586
Assisted vaginal delivery	1 (2.5%)	4 (10%)	5 (6.25%)	
Emergency LSCS	20 (50%)	17 (42.5%)	37 (46.25%)	
Elective LSCS	1 (2.5%)	2 (5%)	3 (3.75%)	
Total	40 (100%)	40 (100%)	80 (100%)	

The mean period of gestation at delivery was significantly lower in the SGA group (37.68 ± 1.15 weeks) compared to the AGA group (38.3 ± 0.99 weeks) (p = 0.011) (Table 2). CTG monitoring was performed in 39 SGA fetuses, as one case resulted in intrauterine death. Category 1 (normal) CTG was more common in AGA fetuses (60%) compared to SGA fetuses (25.64%), whereas Category 2 and 3 (non-reassuring and abnormal) patterns were more frequent in the SGA group (Table 3). 

**Table 2:** Comparison of the period of gestation in weeks between SGA and AGA AGA, adequate for gestational age; SGA, small for gestational age

Period of gestation at delivery (weeks)	SGA (n = 40)	AGA (n = 40)	Total	p-Value (independent t-test)
Mean ± SD	37.68 ± 1.15	38.3 ± 0.99	37.99 ± 1.11	0.011
Median (25th-75th percentile)	37.43 (37-38.571)	38.14 (37.679-39.036)	38 (37.107-38.75)	
Range	36-40.43	36.57-40.14	36-40.43	

**Table 3:** Comparison of intrapartum CTG between SGA and AGA AGA, adequate for gestational age; CTG, cardiotocography; SGA, small for gestational age

CTG	SGA (n = 40)	AGA (n = 40)	Total	p-Value (Fisher’s exact test)
Category 1 CTG	10 (25.64%)	24 (60%)	34 (43.04%)	0.003
Category 2 CTG	23 (58.97%)	15 (37.50%)	38 (48.10%)	
Category 3 CTG	6 (15.38%)	1 (2.50%)	7 (8.86%)	
Intrauterine death	1 (2.5%)	0	1 (1.2%)	
Total	39 (100%)	40 (100%)	79 (100)	

Among cesarean deliveries, fetal distress was the most common indication in the SGA group (85.71%), significantly higher than in the AGA group (47.37%) (p = 0.017). Conversely, failure of induction was more common in the AGA group (31.58%) than in the SGA group (4.76%) (p = 0.04). Other indications did not differ significantly between groups (Table 4).

**Table 4:** Comparison of the indication of LSCS between SGA and AGA AGA, adequate for gestational age; CTG, cardiotocography; LSCS, lower segment cesarean section; SGA, small for gestational age

Indication of LSCS	Number of SGA pregnancies that underwent cesarean section (n = 21)	Number of AGA pregnancies that underwent cesarean section (n = 19)	Total	p-Value (Fisher’s exact test and chi-square test)
Fetal distress (Category 3 + 2 CTG)	18 (85.71%)	9 (47.37%)	27 (67.50%)	0.017
Arrest of descent	1 (4.76%)	1 (5.26%)	2 (5%)	1
Failure of induction	1 (4.76%)	6 (31.58%)	7 (17.50%)	0.04
Maternal request	0 (0%)	1 (5.26%)	1 (2.50%)	0.475
Meconium-stained liquor with poor bishop	3 (14.29%)	2 (10.53%)	5 (12.50%)	1
Bad obstetric history	1 (4.76%)	0 (0%)	1 (2.50%)	1

Regarding umbilical cord pH, a significantly higher proportion of neonates in the AGA group had normal pH values (7.15-7.38) compared to the SGA group (87.5% vs. 58.97%, p = 0.009). The SGA group also had a significantly lower mean cord pH (7.17 ± 0.12) than the AGA group (7.24 ± 0.07), indicating more frequent acidemia at birth (p = 0.003) (Table 5).

**Table 5:** Comparison of umbilical cord pH between SGA and AGA AGA, adequate for gestational age; SGA, small for gestational age

Umbilical cord pH	SGA (n = 40)	AGA (n = 40)	Total	p-Value (Fisher’s exact test)
7.15-7.38	23 (58.97%)	35 (87.50%)	58 (73.42%)	0.009
7-<7.15	14 (35.90%)	5 (12.50%)	19 (24.05%)	
<7	3 (7.5%)	0 (0%)	2 (2.53%)	

RR analysis for adverse neonatal outcomes showed that SGA status was associated with a significantly increased need for resuscitation (RR = 10; 95% CI: 1.34-74.51; p = 0.025). Other outcomes, including low APGAR scores, early neonatal death, respiratory distress, meconium aspiration syndrome, hypoxic-ischemic encephalopathy, sepsis, and prolonged NICU stay, showed higher RRs in the SGA group, though these did not reach statistical significance (Table 6).

**Table 6:** RR of various adverse outcomes in SGA as compared to AGA AGA, adequate for gestational age; SGA, small for gestational age

Outcome	RR (SGA vs. AGA)	95% CI	p-Value
Stillbirth	3	0.126-71.512	0.497
Low birth weight	4	2.112-7.577	<0.0001
APGAR score at 1 min <7	2.222	0.940-5.256	0.069
APGAR score at 5 min <7	9.225	0.513-165.846	0.132
APGAR score at 10 min <7	5.125	0.254-103.456	0.287
Need for resuscitation	10	1.342-74.514	0.025
Early neonatal death	3.075	0.129-73.273	0.488
NICU admission	1.333	0.664-2.676	0.418
Respiratory distress in the baby	1.758	0.773-3.997	0.178
Meconium aspiration syndrome	5.128	0.627-41.931	0.127
Hypoxic-ischemic encephalopathy	3.075	0.129-73.273	0.488
Sepsis	3.075	0.129-73.273	0.488
Feed intolerance	5.125	0.254-103.456	0.287
NICU long admission (>7 days)	2.564	0.528-12.441	0.243

Overall, these findings underscore the increased perinatal risk associated with SGA status, despite similar intrapartum management.

## Discussion

FGR remains a major cause of perinatal morbidity and mortality; however, outcomes can improve with timely detection, Doppler monitoring, and appropriate delivery. This study evaluated the utility of the CPR in identifying adverse outcomes in both SGA and AGA fetuses. While many protocols focus primarily on estimated fetal weight, our findings suggest that abnormal CPR, irrespective of fetal size, is associated with adverse outcomes, indicating that blood flow abnormalities, rather than fetal size alone, are key in late-onset FGR. Maternal characteristics, including age, education, socioeconomic status, mode of conception, and booking status, were comparable across both groups and did not show statistically significant differences. Doppler indices revealed no significant differences in MCA and UA pulsatility indices, but CPR was significantly lower in the SGA group, reinforcing its importance as a marker of fetal compromise. These findings support the inclusion of CPR in routine third-trimester screening for both AGA and SGA fetuses, highlighting its predictive value beyond fetal weight. Prior et al. demonstrated that fetal CPR can identify fetuses at high and low risk for subsequent intrapartum compromise and may be used to risk-stratify pregnancies before labor [10].

There was no statistically significant difference in the mode of labor onset between SGA and AGA fetuses with pathological CPR (p = 0.23), indicating that spontaneous and induced labor occurred at similar rates in both groups. Labor induction was common in both SGA (85%) and AGA (80%) fetuses, reflecting proactive management due to fetal concerns. Spontaneous labor was less frequent in SGA fetuses (10% vs. 20% in AGA), and elective cesarean sections occurred only in SGA cases (5%). These findings suggest higher intervention rates in both groups, consistent with the Barcelona protocol, which recommends induction for pathological CPR by 37 weeks. SGA fetuses were delivered at a significantly earlier gestational age than AGA fetuses (37.68 ± 1.15 vs. 38.3 ± 0.99 weeks; p = 0.011). Most SGA deliveries occurred between 37 and 38 weeks, whereas AGA fetuses were more frequently delivered after 38 weeks. This earlier delivery for SGA reflects clinical decisions driven by concerns about fetal growth and well-being, while AGA pregnancies were allowed to continue to 38-39 weeks without compromising outcomes. The interval between diagnosis and delivery was significantly shorter for SGA fetuses (median: two days) compared to AGA fetuses (median: 8 days; p = 0.019), with 77.5% of SGA fetuses delivered within seven days of diagnosis, highlighting the urgency often associated with growth restriction. Ortiz et al. reported that fetuses with low CPR had higher rates of operative delivery for intrapartum compromise with advancing gestational age, particularly at 40 and 41 weeks, though the predictive value of CPR remained stable throughout term for both SGA and AGA [11].

SGA fetuses in our study exhibited a higher incidence of abnormal CTG patterns. Only 25.6% had normal (Category 1) CTGs, compared to 60% in AGA fetuses (p = 0.003). Category 2 and 3 patterns were more common in SGA (CAT 2: 59%, CAT 3: 15.4%) than in AGA (CAT 2: 37.5%, CAT 3: 2.5%), indicating increased risk of fetal compromise. These results mirror those reported by Epplin et al. and support the need for vigilant monitoring in SGA pregnancies [12].

Mode of delivery and pathological CPR

Pathological CPR was associated with a higher incidence of emergency cesarean sections (46%), regardless of fetal size. Mode of delivery did not significantly differ between SGA and AGA groups, with similar rates of vaginal delivery, assisted vaginal delivery, and cesarean section. This aligns with Prior et al., who reported that pathological CPR independently predicts emergency cesarean delivery in both AGA and SGA fetuses, emphasizing CPR over fetal size as a determinant of delivery mode [10]. Cruz-Martínez et al. found an increased risk of cesarean delivery for non-reassuring fetal status in term fetuses with birth weight <10th centile and CPR <5th centile [13].

CTG abnormalities indicating fetal distress were the leading indication for cesarean section in both groups, particularly in SGA fetuses (85.71% vs. 47.37% in AGA, p = 0.017). Emergency cesarean due to persistent Category 3 CTG was three times more common in SGA, although not statistically significant. Failure of induction was significantly more frequent in AGA fetuses (31.58% vs. 4.76%, p = 0.04), suggesting some preventable interventions. Infants with a cerebro-umbilical ratio below the 10th percentile were six times more likely to undergo cesarean delivery for fetal compromise than those with ratios above the 10th percentile [10]. Anand et al. reported that term AGA babies with CPR ≤ 1 had a ninefold increase in cesarean section rates for intrapartum fetal compromise compared to those with normal CPR [14].

SGA neonates had slightly poorer outcomes, including lower live birth rates (97.5% vs. 100%), higher stillbirth rates (2.5% vs. 0%), and lower mean birth weight (2246.75 g vs. 2912.02 g, p < 0.0001). They also had significantly higher rates of low birth weight and acidemia (cord pH <7.15: 35.9% vs. 12.5%, p = 0.009) compared to AGA fetuses with pathological CPR. Morales-Roselló et al. found that AGA fetuses with abnormal CPR had significantly lower UA and venous pH compared to those with normal CPR [15]. Cruz-Martínez et al. reported that abnormal CPR in late-onset SGA fetuses was associated with a significantly higher rate of emergency cesarean delivery for fetal distress in labor (37.8% vs. 20.4%; p < 0.001) and was a better predictor than isolated MCA measurements [13]. Figueras et al. found that 39.3% of late-onset SGA fetuses had abnormal CPR, which correlated with higher rates of fetal distress (79.1% vs. 10.7%; p < 0.001), lower umbilical cord pH (7.17 vs. 7.25; p < 0.001), and higher NICU admissions (11.25% vs. 5.6%; p = 0.03) [16].

Although APGAR scores at one and five minutes were not statistically different, SGA infants trended toward lower scores and had significantly lower mean scores at 5 and 10 minutes. NICU admission was more frequent in SGA neonates (33.33% vs. 25%), primarily due to respiratory distress (82.6%). Resuscitation was significantly more common in SGA neonates (25% vs. 2.5%, p = 0.007). Although not statistically significant, rates of meconium aspiration, hypoxic-ischemic encephalopathy, sepsis, and feed intolerance were higher in SGA.

Limitations

This study has several limitations. It was conducted at a single center with a small sample size (n = 80), which may limit generalizability and reduce statistical power to detect all significant differences. Only pregnancies beyond 36 weeks were included, and the short neonatal follow-up period (seven days) may have missed late-onset complications or longer-term outcomes. Clinicians were not blinded to fetal CPR status, which could have influenced management decisions. The absence of a control group with normal CPR limits the ability to isolate the specific impact of pathological CPR. Variability in clinical practices and the lack of longitudinal CPR trend analysis further constrain the conclusions. Additionally, a multivariable analysis could not be performed due to the limited sample size, preventing adjustment for potential confounders such as gestational age at delivery. Future larger, multicenter studies across varied gestations, with extended follow-up and comprehensive outcome tracking, are needed to validate these findings and clarify the pathophysiological role of pathological CPR.

## Conclusions

This study aimed to determine whether pathological CPR serves as a significant predictor of adverse perinatal outcomes in both SGA and AGA fetuses. Our findings indicate that pathological CPR is associated with distinct perinatal risks in SGA fetuses, including earlier delivery, higher rates of emergency cesarean section for fetal distress, lower cord pH, greater need for neonatal resuscitation, and increased NICU admissions. Notably, although both SGA and AGA fetuses with pathological CPR were managed according to a uniform protocol, the SGA group consistently exhibited worse neonatal outcomes. This suggests that fetal size significantly modifies the impact of CPR abnormalities, with SGA fetuses being more vulnerable to hypoxic insults even in the presence of similar Doppler findings. These results support the growing recognition that CPR abnormalities reflect underlying placental insufficiency and fetal compromise, particularly when coexisting with SGA.

The clinical implications are twofold: first, CPR assessment should remain a cornerstone of third-trimester fetal surveillance, especially for SGA fetuses; second, while pathological CPR in AGA fetuses may warrant increased monitoring, it should not automatically prompt preterm delivery or invasive intervention in the absence of other risk factors, as over-intervention may lead to unnecessary iatrogenic morbidity without proportional neonatal benefit. In summary, pathological CPR serves as a red flag in SGA fetuses, necessitating heightened vigilance and timely intervention, whereas in AGA fetuses, it should be interpreted within the broader clinical context to avoid overtreatment. These findings underscore the importance of individualized decision-making in obstetric care and support the integration of CPR into risk-based fetal surveillance algorithms.
